# The Influence of Social Capital on Youths’ Anti-Epidemic Action in the Field of Epidemic-Preventative Social Distancing in China

**DOI:** 10.3390/ijerph182111155

**Published:** 2021-10-24

**Authors:** Peiwen Guo, Dong Zeng, Haina Yan, Kin-Sun Chan, Yifen Yin

**Affiliations:** 1 School of Management, Guangzhou Xinhua University, Guangzhou 510520, China; guopw@xhsysu.edu.cn; 2 Department of Government and Public Administration, Faculty of Social Sciences, University of Macau, Macau 999078, China; kschan@um.edu.mo; 3 School of Humanities and Social Sciences, Macao Polytechnic Institute, Macao 999078, China; yfyin@ipm.edu.mo; 4 School of Politics and Public Administration, South China Normal University, Guangzhou 510631, China

**Keywords:** anti-epidemic action, social capital, social distance, field, COVID-19, epidemic, social support, China

## Abstract

Social distancing restrictions for COVID-19 epidemic prevention have substantially changed the field of youths’ social activities. Many studies have focused on the impact of epidemic-preventative social distancing on individual physical and mental health. However, in the field of social distancing for epidemic prevention, what are the changes in youth anti-epidemic action and states caused by their interpersonal resources and interactions? Responding to this question by studying the impact of the elements of social capital in youths’ anti-epidemic actions and anti-epidemic states could help identify an effective mechanism for balancing social distancing for effective epidemic prevention and sustainable social-participation development among youth. Bourdieu’s field theory holds that the elements of social capital change with a change in the field. Therefore, we introduced the specific elements of social capital as independent variables and used a multinomal logistic model to analyze and predict the levels of youth anti-epidemic action through an empirical investigation of 1043 young people in Guangdong Province, China. The results show that, first, level of social distancing for epidemic prevention shows differences by occupation status and income level and correlates with social support. Second, social support and social norms play positive roles in promoting youth participation in anti-epidemic activities when social distance is certain. Third, social capital has a significant positive effect on youth social satisfaction and core relationships; however, social trust has a significant negative effect on youth physical and mental health. This study emphasized that social distancing for epidemic prevention is a special social situational state, which is a field where social capital has an impact on the differential changes in the public-participating actions and habitus of youth.

## 1. Introduction

With COVID-19’s characteristics of strong infectivity, potential asymptomatic infection, and high variability, staying at home and social distancing have become the main strategies to reduce the risk of human-to-human transmission during the epidemic [1] However, social distancing blocks or affects interpersonal physical connections, which impacts people’s personal lives, physical and mental health, and freedom of movement [2], such as through significant declines in individual health status and subjective feelings [3,4]. When serious, social distancing can also lead to severe social consequences [5,6]. Therefore, society needs a mechanism that can not only effectively prevent and control the spread of COVID-19 but also reduce the negative impact on daily life caused by “increased distance communication” under the normalized circumstances of the epidemic.

During the anti-epidemic period in China, the strict enforcement of maintaining “social distance” brought great challenges to people’s everyday living conditions. People had to change their daily habits, especially concerning interpersonal communication, and adapt to new social norms. Youth in China cooperated with the government’s anti-epidemic policy with its various unique ways of interpersonal interaction, which has garnered widespread interest. As a generation of active Internet users, the youth can find epidemic information online, including through various social media platforms, to make suggestions for COVID-19 prevention in their communities, enrich community and rural life using network videos and social platforms, and become propagandists and advisers for middle-aged and older adults. By changing their own “habitus,” such as by giving up frequent outdoor activities, eliminating group gatherings, and adapting to a new form of Internet learning, young people have altered their time and space needs for epidemic prevention in China. In the epidemic context, this includes cooperating with and responding to the government’s anti-epidemic policy, which can be regarded as youth social-participation actions.

However, what are the factors that allow young people to cope with the changes brought about by social distancing? Under the social distancing rules, what changes do their interpersonal resources and interaction patterns have on their anti-epidemic state? Responding to these questions by studying the factors that influence youth’s anti-epidemic actions and anti-epidemic status could help identify an effective mechanism for balancing social distancing for effective epidemic prevention and sustainable social participation development among youth.

### 1.1. Literature Review and Research Hypotheses

#### 1.1.1. Social Capital and Social Distance Field of Epidemic Prevention

Bourdieu (1986) and Coleman (1988) successively advanced the concept of social capital in the 1980s, while Granovetter (1973), Lin (2001), and Burt (1992), respectively, developed the concept from the perspectives of relationship strength, relational resources, and social network structure [7,8,9,10,11]. Social capital can be defined as the scarce resources actors obtain through social ties to achieve behavioral goals. It focuses on formal and informal relationships among and within families, community organizations, and governments [12].

However, the discussion of social capital cannot be separated from the specific social situation, such as the COVID-19 epidemic, in what is referred to as “field” by Pierre Bourdieu. “Field,” “habitus,” and “capital” are three important concepts closely related to each other in Bourdieu’s work, “An Invitation to Reflexive Sociology.” A field is conceptualized as the basic analytical unit of social research, which Bourdieu believes is a relatively independent social space with internal logic and rules. A field can be defined as a network or configuration of objective relationships between various positions. Further, Bourdieu defines habitus as the tendency of an actor to form an action strategy through long-term life experience in a specific field; it is the internalized action consciousness. When actors enter a new field, they are often restricted by the field’s rules, and the habitus they form in the old field will prevent them from adapting to their new field. Therefore, when discussing social capital within the context of the epidemic, the individual characteristics, and state changes during the epidemic, we should also include the perspective of the “field.”

Social distancing to prevent the spread of COVID-19 comprises the field of observation in this study. In this specific field, we discuss the relationship between the changes in social capital elements and anti-epidemic action among youth and the impact on their anti-epidemic state. Recent studies have discussed the impact of social distancing on individual social relationships, social networks, and social support during the COVID-19 epidemic [13]. However, there are few in-depth discussions on the relationship between social distancing and social capital during the epidemic. Therefore, the first hypothesis of this study is as follows:

**Hypothesis** **1** **(H1).** *Social distancing is significantly associated with social capital among youth in the epidemic context.*

#### 1.1.2. Social Capital and Youth Anti-Epidemic Action

Bourdieu believes that capital is the key for actors to compete in a field, and the quantity and results of actors’ capital have crucial effects on their position and role in that field. Through the concept of “epidemic prevention social capital,” Bian and his colleagues (2020) discussed the impact of the changes in cohesive and external social capital on the epidemic prevention effect under social distancing conditions, emphasizing that under effective isolation, the higher a family’s epidemic prevention social capital, the better their performance of epidemic prevention social behaviors, and the better the anti-epidemic effect [14]. Anti-epidemic action among youth as social public participation is affected by many factors, such as personal characteristics, educational background, sense of participation, and ability to participate. Social capital is also an important factor that affects social participation. Therefore, to further discuss the relationship between social capital and youth anti-epidemic action, based on the individual characteristics of social capital, the second hypothesis of this study is as follows:

**Hypothesis** **2** **(H2).** *Social capital has a significant positive impact on anti-epidemic action among youth.*

#### 1.1.3. Social Capital and the Youth’s Anti-Epidemic State

Social capital is multidimensional, with different types, and can produce positive and negative externalities [8,15,16]. The impact of social capital on human quality-of-life indicators has been widely supported, such as in physical and mental health (including self-reported health), subjective well-being, and social attitudes. Woolcock (1998) emphasized that social capital is not unconditionally “good” but may have some adverse effects. For example, social capital contributes to the wider spread of infectious diseases through closer person-to-person contact [17]. Therefore, social capital should be optimized rather than maximized.

“Social norms,” “social trust,” “social support,” and “social connection” are the core elements of the concept of social capital [15,18]. How to maintain social distance from others has become a key issue during the public health emergency of the COVID-19 epidemic. Recent research highlights that individuals experienced more loneliness and a decreased sense of friendship, and that increased social support (such as emotional and instrumental support) emerged during social distancing. Further, high levels of social support are associated with a lower likelihood of anxiety and depression [19]. Social distancing impacts individuals’ lives, physical and mental health, and freedom of movement, and requires a certain degree of “personal sacrifice” [2,3,4]. Social norms, however, create expectations of citizenship and social cooperation and promote selflessness and personal sacrifice for the common good of the community [15]. Fukuyama (1997) defines social capital as “the existence of a specific set of informal values or norms shared among a group of members, allowing for cooperation among them” [20]. Social networks can actively motivate individuals to maintain social distancing to conform to social norms when confronted the COVID-19 health threat.

Although social organizations that incorporate some formal or informal relationships can gain access to key social resources (e.g., information and expertise), these social networks are not built spontaneously but constructed through investment strategies oriented toward the institutionalization of group relationships [21]. Therefore, continuous social connection is often needed to obtain social support, while social distancing during the COVID-19 epidemic can damage social connections [22]. However, interpersonal networks within the context of Chinese relational culture have four major characteristics: strong kinship, functional reusability, strong obligation to reward, and a super-stable relationship circle. Thus, the more prominent these characteristics are, and the closer the social ties are, the richer the social support individuals receive [23].

Many studies have focused on the impact of epidemic-preventative social distancing on social connections [22] or the effects on individual physical and mental health [24,25,26]. Advocating for public health policies during the epidemic, such as staying home and social distancing, needs to weigh personal and public interests, which may be affected by individual internal norms and external social influences [27]. Thus, it is necessary to further investigate the impact of social capital on youth’s anti-epidemic state during the social distancing phase of the pandemic. However, most empirical studies regard social capital as a holistic and homogeneous concept and mainly focus on its positive effects. To fill this research gap and study the relationship between youth anti-epidemic action and social capital, we need to further explore how the elements of social capital (i.e., social norms, social connection, social trust, and social support) affect the interpersonal networks and life habitus of youth, thus affecting their anti-epidemic state in the field of social distancing. Therefore, we propose the following hypothesis:

**Hypothesis** **3** **(H3).** *Different elements of social capital have a significant positive impact on youth anti-epidemic state.*

## 2. Methods

### 2.1. Procedure

In this study, the research group members invited young people aged 15–35 in Guangdong Province to fill out questionnaires, which included 28 questions, through group chats and the moments function of Wechat from June 4 to June 11, 2020. We used a simple random sampling method to collect data online via WeChat. Respondents who voluntarily participated in the questionnaire survey gave consent for their data to be used in the research when they participated in the study. In total, 1043 online questionnaires were collected, with an average response time of 5 min (308.49 s). Questionnaires with intentionally wrong or random answers were screened and identified, and 858 valid questionnaires were collected with a recovery rate of 82.5%.

### 2.2. Participants

The National Bureau of Statistics of China regards “youth” to be individuals between 15 and 35 years of age [28]. As a southern province in China, Guangdong Province government has made outstanding contributions to China’s public health during the COVID-19 epidemic in 2020. Therefore, our study selected individuals aged 15−35 years from cities in Guangdong Province, China, as survey participants, and used the simple random sampling method to collect data online via WeChat. This study was approved by the Academic Ethics Committee of Guangzhou Xinhua University (NO. 2021K003). Among all the participants (female = 625, male = 233), 81.6% of those were aged 15–29, and 18.4% were aged 30–35, 92.4% self-reported that they were undergraduate or above.

### 2.3. Scales and Measurement

#### 2.3.1. Measurement of Social Distance

In this study, we measured social distancing according to the social distancing strategy of “Six Sets of Guidelines on Disease Prevention: For General Use, Tourism, Households, Public Places, Public Transport and Home Observation” [29], which was released by the National Health Commission of the People’s Republic of China on 25th January 2020. As shown in Table 1, “social distance” was primarily measured based on two questions—“Maximum duration of staying home during the epidemic period (23 January to 30 March 2020)” (including six items scored from 1–6) and “Frequency of going out to purchase daily necessities during the epidemic period” (including five items scored from 1–5). The higher the score, the longer social distance was maintained. In the reliability test, the reliability coefficient of all social distance items was 0.732.

#### 2.3.2. Measurement of Youth Anti-Epidemic Action

For this study, we developed a scale of social participation, that was divided into “general participation” and “special participation” [30]. Based on the experience summarized in “Fighting COVID-19 China in Action” [31], we designed a multichoice questionnaire around the idea of “What activities did you participate in during the epidemic period (23 January to 30 March 2020)?”, that included seven items, with a score ranging from 0 to 26 (Table 1). We defined the youth’s participation degree (“without participation,” “general participation” or “special participation”) by computing the scores. A higher score represented a higher level of participation in the action, and a score of 5 or less indicated a general level of participation, a score of more than 5 indicated a special level of participation (Table 1).

#### 2.3.3. Measurement of Youth Anti-Epidemic State

This scale, developed by Diener [32] and revised by Bian et al. [14], is used for evaluated the anti-epidemic state among youth across four dimensions: “studying and working state” (2 items) “physical and mental health state” (3 items) “core relationship state” (2 items) and “social satisfaction” (4 items) (Table 1). Studying and working state and physical and mental health state were used as measurement indicators for young people’s subjective states. Social satisfaction mainly measures one’s satisfaction with the government, social organizations or groups, enterprises, and institutions regarding anti-epidemic measures, and examines social attitudes among youth. Core relationship state mainly refers to whether one’s core relationships with family members and good friends have grown closer during the epidemic. Each item is scored on a five-point scale. Higher scores indicate more thought given to future outcomes. For example, the higher the score, the more harmonious the relationship. The reliability coefficient of all items was 0.773, and in the confirmatory factor analysis, the reliability coefficients of the four dimensions were between 0.8 and 0.9, indicating that the measurement scale has good reliability.

#### 2.3.4. Measurement of Social Capital

Social Capital Assessment Tools (SCAT) is the earliest systematic tool for measuring Social Capital, which some scholars have improved upon, and the new system is called (A-SCAT). This study refers to Putnam’s tool for measuring macro social capital, which measures social capital from the dimensions of social network, norms, and trust [15]. Based on the empirical research on the localization of social capital in China [23,33], this study’s questionnaires were designed to measure social capital from the dimensions of social support (2 items), social norms (3 items), social connection (3 items), and social trust (2 items). Combined with the characteristics of social distancing policy in China, a special scale for measuring social capital was designed for this study (Table 1). To examine the support young people received during the epidemic period, according to the definition of social support, which refers to the size, density, reciprocity of one’s social network versus the availability of certain types of aids including practical and emotional support [34,35], this study mainly measured social support as “the use of informal networks (social support networks)” and “resources flowing in social networks” [33]. Since the outbreak of the epidemic at the beginning of 2020, “wearing a mask,” “washing your hands,” “not spreading misinformation” has become the behavioral norm for people in China to cooperate with the government in the implementation of epidemic prevention measures and social distancing. Thus, these factors were used to measure social norms in our study. Social connection was measured by online interactions between young people and their families and friends, and social trust was measured as the level of trust young people had in different epidemic information sources (Table 1). Each item is scored on a five-point scale, from 1 = very unlike me to 5 = very like me. Higher scores indicate more social capital elements. The reliability coefficients for all social capital items were greater than 0.6 (Cronbach’s alpha = 0. 806), indicating that the scale’s internal reliability was relatively high.

#### 2.3.5. Measurement of Occupation Status and Monthly income

For occupation status, this study referred to the occupation indicator of individual socioeconomic characteristics in Bian’s study [23], which was used to develop Lin Nan’s social resources theory [10]. We made some modifications to Bian’s scale, according to the latest national occupation division by the National Bureau Statistics of China [28] (Table 1), including seven groups valued from 1 to 7. The higher the value, the higher the occupation status. In addition, based on the division of the monthly income level from the National Bureau Statistics of China in 2019 [28], income was divided into five groups. The higher the value, the higher the income level.

### 2.4. Statistical Analysis

First, we used confirmatory factor analysis (CFA) to test the theoretical construction dimension of social capital in the field of epidemic-preventiative social distancing. Second, this study explored the association between social distance and several elements of social capital among youth with various individual characteristics (e.g., gender, education, occupation status and income indicators) by using correlation analysis for testing hypothesis 1. Third, we used a multinomial logistic regression model and the stepwise method to test hypothesis 2, exploring the impact of social capital on youths’ anti-epidemic actions in the field of epidemic-preventative social distancing. Finally, the study tested hypothesis 3 through multiple linear regression, investigating the impact of social capital on youths’ anti-epidemic states. In this study, SPSS 21.0 (IBM, New York, NY, USA) was used for the descriptive statistical analysis, analysis of variance, correlation analysis and regression analysis. The CFA was conducted using SPSS AMOS 22.0 (IBM, New York, NY, USA).

## 3. Results

### 3.1. Factor Analysis of Social Capital

To test the fit between the overall measurement model of social capital and the sample data, AMOS was used for confirmatory factor analysis (CFA). The parameters of the four-factor model showed that the model’s goodness of fit was as follows: chi-squared = 208.591, *p* = 0.000 < 0.05; CFI = 0.995 > 0.9, TLI = 0.986 > 0.9, RMSEA = 0.030 < 0.05 [36,37]).

**Figure 1:**
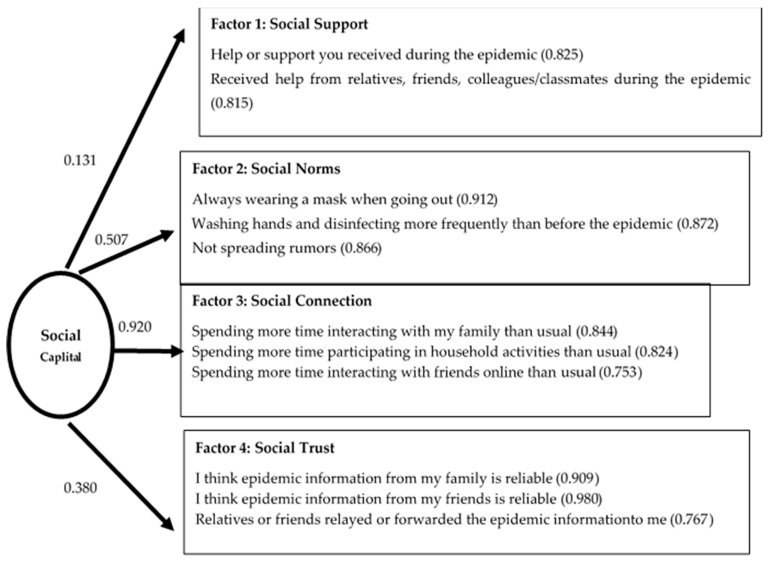
Factor analysis results for social capital.

As shown in Figure 1, the four factors of social capital were independent of each other, among which social norms (0.507) and social connection (0.920) had the highest degree of explanation, followed by social trust (0.380), and then social support (0.131) with the weakest degree of explanation. For social support, the factor loadings of the two variables were both higher than 0.8 (0.825, 0.815), which indicated that the information or support youth obtained during the epidemic period mainly came from core relationships, such as family members and friends. Similarly, the factor loading values of the explanatory variables of social norms (0.912, 0.872, 0.866), social connection (0.844, 0.824, 0.753), and social trust (0.909, 0.980, 0.767) were high, which indicated that these variables could strongly explain the relevant dimensions of social capital. The results of factor analysis indicated that the specific connotation of the concept of social capital among youth during the epidemic period was mainly embodied in social norms, social connection, and social trust. Thus, youth were better able to achieve behavioral goals during the epidemic period when they better abided by the social norms of “wearing a mask, washing hands frequently, not spreading rumors,” interacted more with family members and good friends online, and trusted the information obtained from their core relationships.

### 3.2. Preliminary Analysis

#### 3.2.1. Descriptive Statistics for Youth Anti-Epidemic Action, Social Distance, and Social Capital during the Epidemic

As shown in Table 2, anti-epidemic participation among youth was mainly at the level of general participation (*N* = 572, 67%), which was in response to the government’s call to engage in staying at home and cooperating with anti-epidemic action. Social distance was mainly measured by the maximum duration of staying at home and frequency of going out, with an average value of 7.377, which was a high level, indicating that young people could maintain a relatively long period of social distancing during the epidemic. Regarding social capital, the mean values for social norms (13.89), social connection (16.14), and social trust (10.78) were high, and there were no significant differences among individuals, indicating that youth could observe the social norms of epidemic prevention, such as wearing a mask, washing their hands frequently, and not spreading rumors. Interactions with family at home and online communication with core relationships were more centralized and stable, and youth reported a higher level of trust as the epidemic information was shared between family members and friends, which was consistent with the strong relationship culture in China. However, the level of social support was relatively low (mean = 3.73), and the individual difference was large (SD = 1.892), which will be discussed in more detail below.

#### 3.2.2. Descriptive Statistics for Youth Anti-Epidemic State and Individual Characteristics

Table 2 indicates that the overall physical and mental health state of the sample was good during the epidemic. Similarly, youth had higher social satisfaction with the arrangements for implementing epidemic prevention measures in communities, schools, or enterprises (mean = 16.12, SD = 3.111). The state of core relationships with family members and good friends was also good (mean = 7.93, SD = 1.731). Notably, the studying and working state of youth was at a low level (mean = 5.56), and individual differences were large (SD = 2.13). This indicated that young people’s studying and working state was greatly affected during the epidemic period and that the degree of variation was high. The sample’s income level (mean = 3.47, SD = 1.651) and occupation status (mean = 3.11, SD = 1.85) were at an average level, and because income level and occupation status were sequential variables and the standard deviation was greater than 1, individual differences in the sample were relatively large.

### 3.3. The Association between Social Capital and Social Distancing in the Epidemic Field

We analyzed the association between social capital and social distance among youth with various individual features by using correlation analysis (Table 3). As shown in Table 3, the results of the correlation analysis partially supported Hypothesis 1: Social distance was positively correlated with social support (β = 0.068, *p* = 0.045) and was not significantly correlated with other elements of social capital. Social distance showed a significant correlation with gender, age, occupation status, and income level. Social support (β = 0.113, *p* = 0.001) and social trust (β = −0.078, *p* = 0.045) under social capital showed significant correlation with educational level.

### 3.4. Model Analysis Results

Table 4 shows the regression analysis results for the effects of social capital on youth anti-epidemic actions and anti-epidemic state. The dependent variable in Model 1 was youth anti-epidemic action. In Model 1, youth anti-epidemic action was an ordinal variable (valued from 0 to 26), representing different degrees of youth participation in anti-epidemic actions. To explore the influencing factors of differences in social participation action among the youth, we first converted the values of “without participation” from 0 points to 1 (1 = “without participation”), “general participation” from 2–5 points to 2 (2 = “general participation”), “special participation” from more than 5 points to 3 (3 = “special participation”). We then used a multinomial logistic regression model and the stepwise method, using the element variables of social distance and social capital as the mandatory input items and the individual characteristics variables as the stepwise items to construct the model equation. According to the descriptive statistics, participation action among youth during the epidemic was mainly at the level of general participation; thus, this was set as the reference category for the dependent variable. Based on the model fit, the *p*-value of the likelihood ratio test was less than 0.05, and the significance test of the regression equation was passed, which shows that the model was reasonable. Table 4 shows the results of this analysis.

The dependent variable of Models 2 to 9 was the effects of youth anti-epidemic action, that is, youth anti-epidemic state, which was measured from four dimensions: studying and working state, physical and mental health state, social satisfaction, and core relationship state. A group of nested models was used for each dependent variable to test whether social capital had an independent effect on youth anti-epidemic state in the field of social distance.

#### 3.4.1. Impact of Social Capital on Youth Anti-Epidemic Action in the Field of Epidemic Social Distance

The variables of age and monthly income as stepwise were deleted, indicating that they were not suitable to add to the equation model. The results of the multinomial logistic regression model analysis for Model 1 showed that the likelihood-ratio test indices for social distance (LRC = 6.165, *p* = 0.046), social support (LRC = 35.539, *p* = 0.000), educational level (LRC = 11.452, *p* = 0.003), and occupation (LRC = 7.724, *p* = 0.021) were significant, indicating that these four variables added to the model had an effect on youth anti-epidemic action. The regression equations constructed using the stepwise method are as follows:

Log (P|without participating) = 1.933 0.094 ∗ social distance − 0.244 ∗ social support − 0.131 ∗ social norms + 0.085 ∗ social connection + 0.068 ∗ social trust − 0.752 ∗ education − 0.036 ∗ occupation (1)

Log (P|special participation) = −6.090 + 0.067 ∗ social distance + 0.198 ∗ social support + 0.026 ∗ social norms + 0.052 ∗ social connection − 0.003 ∗ social trust + 0.342 ∗ education + 0.115 ∗ occupation (2)

Regression Equation (1) shows that, compared to the general participation level among youth, the lower the social support, social norms, educational level, and occupation state, the greater the chance young people would not participate in anti-epidemic action. Social connection and social trust increased the probability of nonparticipation by 0.085 and 0.068 units, respectively; however, the difference was not statistically significant. When social distance and social capital were constant, the higher the educational level and occupation status, the higher the probability that youth would not participate in anti-epidemic action at a general participation level (e.g., home isolation), for which educational level was statistically significant.

From Equation (2), when social distance, educational level, and occupation status were constant, obtaining social support, abiding by social norms, and maintaining social connections could increase the probability of youth engaging in general participation to special participation by 0.198, 0.026, and 0.052 units, respectively. Among these, social support was statistically significant. Social trust reduced the probability of the corresponding special participation action by 0.003 units, which was not statistically significant. Similarly, the higher the educational level and occupational status, the higher the probability of special participation, for which occupational status was statistically significant.

Model 1 partly supported Hypothesis 2, and the results showed that maintaining social distance during the epidemic increased the probability of general participation in anti-epidemic actions (e.g., staying at home) and special social participation (e.g., volunteer activities). When social distance was constant, the effect of social capital on youth anti-epidemic participation action varied according to different elements. Social support and social norms had significant positive effects on youth action from nonparticipation to general participation, especially the effect of social support on the probability of special participation being more significant. Compared to general participation, social connection increased the probability of youth not participating in anti-epidemic action and participating in special anti-epidemic action; however, this was not significant. In addition, regarding individual characteristics, educational level had a significant positive effect on promoting youth from nonparticipation to general participation, while occupational status, as an individual characteristic of social stratification with a stable positive correlation with social capital, had a significant positive effect on increasing the probability of special participation among youth. Thus, under the social distancing conditions of the epidemic situation, different elements of social capital had differing effects on youth anti-epidemic action.

#### 3.4.2. Impact of Social Capital on Youth Anti-Epidemic State in the Field of Epidemic Social Distance

To identify a mechanism that will not only ensure the effects of epidemic prevention but also allow youth to maintain a healthy social life, we used Models 2 to 9 to explore the role that social capital played on the normalization of the epidemic situation by analyzing the influence of social distance, social capital, and anti-epidemic action on youth anti-epidemic state. As shown in Table 4, the impact of social distancing on the life habitus of young people during the epidemic was not significant. The $R^{2}$ values of Models 3, 5, 7, and 9 were gradually enhanced and significant after adding the elements of social capital, and the explanatory power of the models was gradually enhanced, indicating that social capital has a significant impact on youth anti-epidemic state in the social distancing field. Different social capital elements have differing effects on varying anti-epidemic states, which supported Hypothesis 3.

In addition, monthly income and educational level had an independent impact on the effect of youth anti-epidemic action. The higher the monthly family income, the better the working and learning state, as well as the physical and mental health state, among youth. Notably, the higher young people’s educational level was, the lower their social satisfaction with “prevention and control measures in the community,” or “arrangements for stopping classes,” and “arrangements for resuming work and classes.”

## 4. Discussion

Bourdieu believes that analysis of an actor and their behavior not only needs to start from the macrolevel social environment but also needs to understand the actor’s field and its capital and habitus. Analyzing the habitus of the actor in the field can clearly show how various forms of capital contend with each other and can also explore the reasons behind the actors’ behavior. Therefore, this study aimed to empirically explore the social distancing field of anti-epidemic action, and how different social capital factors would affect youth anti-epidemic behavior and habitus. According to the analysis of survey data, young people in the main cities of Guangdong could maintain social distancing for a long time during the epidemic period, and social distancing among youth showed significant social class differences in occupational status and income. Regarding social distancing for epidemic prevention, the main performance of young people’s anti-epidemic action was the general participation in staying at home and cooperating with anti-epidemic policies.

Youth demonstrated an overall good anti-epidemic state; however, there were some individual differences. By introducing the specific elements of social capital, we found that social support and social norms had significant positive effects on young people’s participation in the anti-epidemic campaign, while their habitus of life was also influenced by different elements of social capital during the epidemic period.

### 4.1. Analysis of Hypotheses 1 and 2

Bourdieu posits that the amount and structure of the capital that actors own can determine their position in the field, and the rank of that capital will vary with changes in the field [7]. We found that social distancing under epidemic prevention and control among youth showed significant differences according to occupation, education and income level and was significantly positively correlated with social support. However, the direct effect of social distance on youth action and anti-epidemic state was not significant. Many studies have shown a stable positive correlation between people’s social capital and their education, occupation, and income [23]. Therefore, the social class differences in social distancing reflect not only the differences in youth social capital but also the prominent role of different elements of social capital in this field.

Moreover, our analysis found that in the case of epidemic social distancing, in addition to occupation status, social support and social norms play a positive role in promoting youth participation in anti-epidemic activities. When young people can obtain information about the epidemic situation from various channels of formal networks, such as the government and community, they can cooperate with home epidemic prevention, social distancing, and other anti-epidemic actions. Additionally, as the main channel of “human resources,” strong ties in informal networks have personal inclusiveness. Specifically, they show understanding and tolerance of social distancing and less social interaction during the epidemic period but still provide relevant human resources (e.g., material support for epidemic prevention and spiritual support).

### 4.2. Hypothesis 3 Was Verified by the Regression Analysis of Youths’ Anti-Epidemic State

The regression analysis of youth’s anti-epidemic state in this study is actually the analysis of actors’ habitus in the social distancing field; it can clearly show how various actors’ social capital contends though exploring the effects of the social capital and the anti-epidemic action on the anti-epidemic state, and could comprehensively explain the mechanism of youth anti-epidemic action. Based on the analysis results, the discussion points are as follows.

First, social capital had a significant positive effect on social satisfaction and core relationship state among youth. The results showed that the social norms of “wearing a mask, washing hands frequently, and not spreading rumors,” social support from family and friends, social trust in family interactions and online communication, and sharing epidemic information could enhance social satisfaction and the harmony of core relationships among young people. Owing to the popularization of information network technology in China and the timely disclosure of epidemic information by the government, information resources were disseminated overall through family interactions and social links on online exchanges in informal networks (core relationships). This improved young people’s social trust in epidemic information and motivated them to adhere more closely to social norms, thereby enhancing the effect of epidemic prevention. However, the long duration of staying home for epidemic prevention led to young people spending more time with their families, although they could not work or study normally, and increasing their online contact between relatives and friends. In this case, social distancing generally did not affect social contact, which not only promoted young people’s cooperation with the government’s epidemic prevention policies but also strengthened their core relationships. Therefore, the epidemic prevention effect improved. This is in line with the positive role of social capital in general: the intake and mobilization of social resources to enhance effective behavior and obtain better social support. This shows that during the epidemic period, strong ties may have been more inclusive—this helps maintain a harmonious relationship without social activities such as meetings and gatherings besides providing social support for young people to fight against the epidemic. It can be seen that China’s strong relationship culture has played an important role during the COVID-19 epidemic.

Second, social trust in social capital had a significant negative impact on the physical and mental health of young people. In this study, social trust was mainly measured by the trust of youth in epidemic information conveyed between family and friends, and epidemic information may cause people to experience a certain degree of panic, anxiety, and other negative emotions, which is in line with previous studies [38,39]. Therefore, social trust can also have a certain negative impact on physical and mental health among youth.

Third, youth anti-epidemic action had a significant negative impact on physical and mental health. Young people’s participation in anti-epidemic activities was generally manifested as staying home for epidemic prevention, cooperating in epidemic prevention and control measures, and other general participation activities. Although young people spent more time interacting with their families, they also reduced the time spent during normal social interaction (e.g., gatherings), which inevitably had a negative impact on their physical and mental health, such as anti-epidemic fatigue, decreasing anti-epidemic action, and depressive symptoms. The effects of anti-epidemic action on the studying and working state and social satisfaction among youth was not significant. Due to online teaching and office work applied via the government advocacy of “suspended class, ongoing learning” and “orderly resumption of work and production”, young people could not only stay at home to prevent the spread of the epidemic but also achieve a better balance with work and study.

## 5. Conclusions

The emergence of social distance in epidemic prevention can be seen as a change in the original activity field of youth, which changes not only the geographical field but also social network links and relationships, action rules, and resource support. This study emphasized that the social class differences in social distancing reflect not only the differences in youth social capital but also the prominent role of different elements of social capital in this field.

The social distancing restrictions of home epidemic prevention have substantially changed the original field of youth social activities, and should be a major challenge for youth social interaction. However, our research showed that youth cooperation with and participation in home epidemic prevention is at very high levels. The long-term social support model dominated by strong ties (e.g., material support for epidemic prevention and spiritual support) played an important role in the youth’s anti-epidemic action and social satisfaction. The anti-epidemic actions and the evenly distributed access to epidemic information have had different degrees of negative effects on the youth’s physical and mental health. However, strict and effective epidemic prevention guidelines, the reconstruction of social order by social norms and social connections based on the Internet and networks can compensate for the discomfort brought by social distancing and anti-epidemic actions.

Therefore, social distancing for epidemic prevention is a special, social, situational state, and it is a field where social capital has an impact on the differential changes in public participation actions and habitus of youth. This study will help further explain the behavioral choices of youth regarding combating the epidemic, and even participating in public policy.

## 6. Limitations and Future Direction

This study discussed how the social capital in the specific field of “social distance” affected the youth’s anti-epidemic action and its state under the background of Chinese anti-epidemic policy. Some limitations of this study should be taken into account when interpreting our findings. First, owing to the strict anti-epidemic policy requirements at the time in China, it was difficult to control the structure of sample data through online questionnaires via social media. Second, because of the differential implementation of epidemic prevention and control measures among cities in Guangdong Province, many other factors may have occurred at the individual level and regional level. What can be tracked and studied in the future is what changes will take place in these social capital elements in the field of the new normalization of epidemic prevention and control, and what impact will it have on youths’ value judgment, social participation, action strategies and life habitus.

## Figures and Tables

**Table 1:** Scales and Measurements.

	Variables	Item	Range of Measured Values	Cronbach’s Alpha
Social Distance		Maximum duration of staying home during the epidemic period	1 = “<1 week” 2 = “1 week” 3 = “1–2 weeks” 4 = “2 weeks” 5 = “2–3 weeks” 6 = “3 weeks or more”	0.732
		Frequency of going out to purchase daily necessities during the epidemic period	1 = “3 times or more per week” 2 = “2–3 times per week” 3 = “2 times per week” 4 = “1–2 times per week” 5 = “≤1 time per week”	
Anti-epidemic Action		What activities did you participate in during the epidemic period (January 23 to 30 March 2020)?	0 = without participation 2 = Stayed home 3 = Cooperated with anti-epidemic policy actions 4 = Assisted in combating the epidemic 5 = Organized anti-epidemic activities 6 = Participated in voluntary anti-epidemic activities 6 = Made donations	--
Anti-Epidemic State Cronbach’s Alpha = 0.773	Studying and working state	Less efficient at school/work than usual	From 1 = very like me to 5 = very unlike me	0.926
		Difficulties in concentrating on work/study		
	Physical and mental health state	Emotional fluctuations (e.g., anxiety, worry, and fear)	From 1 = very like me to 5 = very unlike me	0.830
		Feeling stressed		
		Dizziness, accelerated heartbeat, loss of appetite, and other physical discomfort may occur		
	Core Relationship State	Relationship with family is more harmonious than usual.	From 1 = very unlike me to 5 = very like me	0.847
		Relationship with friends is more harmonious than usual.		
	Social Satisfaction	Satisfied with prevention and control measures, such as “setting-up checkpoints” for registration and temperature measurement in the community/village street	From 1 = very unlike me to 5 = very like me	0.904
		Satisfied with the disinfection of public areas in the community/village street		
		Satisfied with the relevant arrangements for schools and other educational institutions for implementing “suspended class, ongoing learning”		
		Satisfied with the arrangements of enterprises/schools for resuming work/study		
	Social Support	Help or support you received during the epidemic	From 1 = very unlike me to 5 = very like me	
		Received help from relatives, friends, colleagues/classmates during the epidemic		0.603
Social Capital	Social Norms	Always wearing a mask when going out	From 1 = very unlike me to 5 = very like me	
Cronbach’s Alpha = 0.806		Washing hands and disinfecting more frequently than before the epidemic		0.901
		Not spreading rumors		
	Social Connection	Spending more time interacting with my family than usual	From 1 = very unlike me to 5 = very like me	
		Spending more time participating in household activities than usual		0.818
		Spending more time interacting with friends online than usual		
	Social Trust	I think the epidemic information from my family is reliable	From 1 = very unlike me to 5 = very like me	
		I think the epidemic information from my friends is reliable		0.912
		Relatives or friends relayed or forwarded the epidemic information I received		
Individual Characteristics	Monthly Income	What is your average monthly income?	1 = CNY ≤2000 2 = CNY 2000−5000 (=5000) 3 = CNY 5000−10,000 (=10,000) 4 = CNY 10,000−20,000 (=20,000) 5 = CNY >20,000	--
	Occupation Status	Which option is the closest description of your occupation type?	7 = Heads of state organisation/party/enterprise 6 = Personnel in a specific technical field 5 = Staff of public administration, enterprise or institution 4 = Business and service personnel 3 = Production personnel in agriculture, forestry, animal husbandry, fishery or water conservancy 2 = Others (e.g., students) 1 = Unemployed	--

**Table 2:** Statistical Analysis of Variable Description.

Variable	Sample Size	Range of Measured Values	Mean Value	Standard Deviation
Social Distance	858	2−11	7.377	2.511
Social Capital				
Social Support	858	0−11	3.732	1.892
Social Norms	858	3−15	13.89	2.168
Social Connection	858	4−20	16.14	3.266
Social Trust	858	3−15	10.78	2.509
Anti-epidemic Action				
Without Participation	61	0	4.094	3.726
General Participation	572	1−5		
Special Participation	225	6−26		
Anti-epidemic State				
Studying or Working State	858	2−10	5.56	2.130
Physical and Mental Health State	858	3−15	9.93	2.899
Social Satisfaction	858	4−20	16.12	3.111
Core Relationship State	858	2−10	7.93	1.731
Individual Characteristic				
Monthly Income	858	1–5	3.47	1.651
Occupation Status	858	1–7	3.11	1.849

**Table 3:** Correlation Coefficient Matrix of Social Capital and Social Distance During the Epidemic.

Variables	1	2	3	4	5	6	7	8	9	10
1. Social Distance	1									
2. Social Support	0.068 *	1								
3. Social Norms	0.016	0.062	1							
4. Social Connection	0.027	0.117 **	0.467 **	1						
5. Social Trust	−0.048	0.107 **	0.183 **	0.349 ***	1					
6. Gender	0.119 **	−0.026	0.081 *	−0.036	−0.036	1				
7. Age	−0.209 **	−0.061	0.035	0.002	−0.010	0.060	1			
8. Educational level	0.082 *	0.113 **	−0.025	−0.054	−0.078 *	0.043	−0.123 **	1		
9. Occupation status	−0.190 **	0.001	−0.008	0.020	0.062	−0.086 *	0.247 **	0.008	1	
10. Income level	−0.273 **	−0.043	0.019	0.018	−0.021	−0.045	0.357 **	−0.017	0.615 **	1

Note: * *p* < 0.05, ** *p* < 0.01, *** *p* < 0.001.

**Table 4:** Regression Analysis of Youth Anti-Epidemic Action and Its Effects.

Variable	Anti-Epidemic Social Participation Action (Model 1)		Studying and Working State		Physical and Mental Health State		Social Satisfaction		Core Relationship State	
	Without Participation	Special Participation	Model 2	Model 3	Model 4	Model 5	Model 6	Model 7	Model 8	Model 9
Social Distance	−0.094	0.067	−0.013	−0.012	0.051	0.041	0.020	0.018	0.024	0.007
Social Capital										
Social Support	−0.244 ***	0.198 ***	--	0.034	--	0.101	--	0.112 *	--	−0.035
Social Norms	−0.131 *	0.026	--	−0.062	--	−0.020	--	0.243 ***	--	0.087 ***
Social Connection	0.085	0.052	--	−0.037	--	−0.002	--	0.208 ***	--	0.362 ***
Social Trust	0.068	−0.003	--	−0.007	--	−0.124 **	--	0.384 ***	--	0.064 ***
Anti-epidemic Participation Action	--	--	−0.003	−0.003	−0.061 *	−0.068 *	0.020	−0.023	0.026	0.005
Individual Characteristics										
Age	--	--	0.162	0.169	0.257	0.260	0.219	0.268	0.088	0.108
Education	−0.752 **	0.342	−0.107	−0.144	0.170	0.069	−0.898 ***	−0.633 **	−0.367 **	−0.142
Monthly income	--	--	0.181 **	0.185 **	0.183 *	0.170 *	0.032	0.074	0.035	0.025
Occupation	−0.036	0.115 **	0.053	0.052	0.066	0.084	0.122	0.063	0.055	0.045
Constant	1.933	−6.090	4.947	6.468	7.725	9.488	18.595	6.269	8.635	0.351
$R^{2}$	--	--	0.036 ***	0.048 ***	0.024 **	0.038 ***	0.024 **	0.308 ***	0.020 **	0.614 ***
Model fit of Model 1: -2LL chi-squared statistic = 1306.230 (79.711), DF = 14, *p* = 0.000; Nagelkerke $R^{2}$ = 0.111										

Note: 1. Model 1 is multinomial logistic regression; Models 2–9 are multiple linear regression. 2. The reference category for the dependent variable in Model 1 is “2 = General Participation”. 3. The variables of age and monthly income as stepwise were deleted. 4. * *p* < 0.05, ** *p* < 0.01, *** *p* < 0.001.

## Data Availability

The data presented in this study are available on request from the corresponding author.

## References

[1] Sajed A.N., Amgain K. (2020). Corona Virus Disease (COVID-19) Outbreak and the Strategy for Prevention. Eur. J. Med. Sci..

[2] Kaufman K.R., Petkova E., Bhui K.S., Schulze T.G. (2020). A global needs assessment in times of a global crisis: World psychiatry response to the COVID-19 pandemic. BJPsych Open.

[3] Ripon R.K., Mim S.S., Puente A.E., Hossain S., Babor M.H., Sohan S.A., Islam N. (2020). COVID-19: Psychological effects on a COVID-19 quarantined population in Bangladesh. Heliyon.

[4] Cerbara L., Ciancimino G., Crescimbene M., La Longa F., Parsi M.R., Tintori A., Palomba R. (2020). A nation-wide survey on emotional and psychological impacts of COVID-19 social distancing. Eur. Rev. Med. Pharmacol. Sci..

[5] Lippi G., Henry B.M., Bovo C., Sanchis-Gomar F. (2020). Health risks and potential remedies during prolonged lockdowns for coronavirus disease 2019 (COVID-19). Diagnosis.

[6] Khan A.G., Kamruzzaman, Rahman N., Mahmood M., Uddin A. (2021). Quality of life in the COVID-19 outbreak: Influence of psychological distress, government strategies, social distancing, and emotional recovery. Heliyon.

[7] Bourdieu P., Richardson J. (1986). The forms of capital. Handbook of Theory and Research in the Sociology of Education.

[8] Coleman J.S. (1988). Social capital in the creation of human capital. Am. J. Sociol..

[9] Granovetter M.S. (1973). The Strength of Weak Ties. Am. J. Sociol..

[10] Lin N. (2001). Social Capital: A Theory of Social Structure and Action.

[11] Burt R. (1992). Structural Holes: The Social Structure of Competition.

[12] Putnam R.D. (1993). Making Democracy Work: Civic Traditions in Modern Italy.

[13] Campbell A.D. (2020). Practical implications of physical distancing, social isolation, and reduced physicality for older adults in re-sponse to COVID-19. J. Gerontol. Soc. Work..

[14] Bian Y., Ma X., Guo X., Miao X., Lu X. (2020). Theoretical construction and behavioral significance of virus-combat social capital. J. Xi’an Jiaotong Univ. (Soc. Sci.).

[15] Putnam R.D. (2001). Social capital: Measurement and consequences. Can. J. Policy Res..

[16] Woolcock M. (1998). Social capital and economic development: Toward a theoretical synthesis and policy framework. Theory Soc..

[17] Holtgrave D.R., Crosby R.A. (2003). Social capital, poverty, and income inequality as predictors of gonorrhoea, syphilis, chlamydia and AIDS case rates in the United States. Sex. Transm. Infect..

[18] Jha A. (2019). Financial Reports and Social Capital. J. Bus. Ethic.

[19] Gariépy G., Honkaniemi H., Quesnel-Vallée A. (2016). Social support and protection from depression: Systematic review of current findings in Western countries. Br. J. Psychiatry.

[20] Fukuyama F. (1997). Social capital and the modern capitalist economy: Creating a high trust workplace. Stern Bus. Mag..

[21] Portes A. (1998). Social Capital: Its Origins and Applications in Modern Sociology. Annu. Rev. Sociol..

[22] Bierman A., Upenieks L., Schieman S. (2021). Socially Distant? Social Network Confidants, Loneliness, and Health during the COVID-19 Pandemic. Soc. Curr..

[23] Bian Y., Zhang W. (2001). Economic systems, social networks and occupational mobility. Soc. Sci. China.

[24] Clemens V., Deschamps P., Fegert J.M., Anagnostopoulos D., Bailey S., Doyle M., Eliez S., Hansen A.S., Hebebrand J., Hillegers M. (2020). Potential effects of “social” distancing measures and school lockdown on child and adolescent mental health. Eur. Child Adolesc. Psychiatry.

[25] El-Zoghby S.M., Soltan E.M., Salama H.M. (2020). Impact of the COVID-19 Pandemic on Mental Health and Social Support among Adult Egyptians. J. Community Health.

[26] Zhu Y., Zhang L., Zhou X., Li C., Yang D. (2021). The impact of social distancing during COVID-19: A conditional pro-cess model of negative emotions, alienation, affective disorders, and post-traumatic stress disorder. J. Affect. Disord..

[27] Coroiu A., Moran C., Campbell T., Geller A.C. (2020). Barriers and facilitators of adherence to social distancing recommendations during COVID-19 among a large international sample of adults. PLoS ONE.

[28] National Bureau of Statistics of China (2019). China Statistical Yearbook. http://www.stats.gov.cn/tjsj/ndsj/2019/indexch.htm.

[29] National Health Commission of the People’s Republic of China Six Sets of Guidelines on Disease Prevention: For General Use, Tourism, Households, Public Places, Public Transport and Home Observation. http://www.nhc.gov.cn/xcs/kpzs/202001/ce1f24ef099745cbbc251dcb4295e547.shtml.

[30] Kim D., Kawachi I. (2006). A Multilevel Analysis of Key Forms of Community- and Individual-Level Social Capital as Predictors of Self-Rated Health in the United States. J. Hered..

[31] The State Council Information Office of the People’s Republic of China Fighting COVID-19 China in Action. https://baijiahao.baidu.com/s?id=1668913001492011118&wfr=spider&for=pc.

[32] Diener E. (2000). Subjective well-being: The science of happiness and a proposal for a national index. Am. Psychol..

[33] Zhao Y., Luo J. (2005). How to measure social capital: A review of empirical research. Soc. Sci. Abroad.

[34] Cohen S., Wills T.A. (1985). Stress, social support, and the buffering hypothesis. Psychol. Bull..

[35] Lakey B., Cronin A., Dobson K., Dozois D. (2008). Low social support and major depression: Research, theory and methodological issues. Risk Factors in Depression.

[36] Bollen K., Long J.S. (1993). Testing Structural Equation Models.

[37] Hu L., Bentler P.M. (1999). Cutoff criteria for fit indexes in covariance structure analysis: Conventional criteria versus new alternatives. Struct. Equ. Model..

[38] Gao J., Zheng P., Jia Y., Chen H., Mao Y., Chen S., Wang Y., Fu H., Dai J. (2020). Mental health problems and social media ex-posure during COVID-19 outbreak. PLoS ONE.

[39] Geirdal A.Ø., Ruffolo M., Leung J., Thygesen H., Price D., Bonsaksen T., Schoultz M. (2021). Mental health, quality of life, wellbeing, loneliness and use of social media in a time of social distancing during the COVID-19 outbreak. A cross-country comparative study. J. Ment. Health.

